# Eco-Friendly Coal Gangue and/or Metakaolin-Based Lightweight Geopolymer with the Addition of Waste Glass

**DOI:** 10.3390/ma16176054

**Published:** 2023-09-03

**Authors:** Celina Ziejewska, Agnieszka Bąk, Krzysztof Hodor, Marek Hebda

**Affiliations:** 1 Faculty of Materials Engineering and Physics, Cracow University of Technology, Warszawska 24, 31-155 Cracow, Poland; celina.ziejewska@pk.edu.pl (C.Z.); agnieszka.bak@pk.edu.pl (A.B.); 2 NETZSCH (Netzsch Instrumenty Sp. z o.o.), Halicka 9, 31-036 Cracow, Poland; krzysztof.hodor@netzsch.com

**Keywords:** micro-computed tomography (microCT), TG-FTIR, thermal conductivity, foam, porosity

## Abstract

Massive amounts of deposited coal gangue derived from the mining industry constitute a crucial problem that must be solved. On the other hand, common knowledge about the recycling of glass products and the reuse of waste glass is still insufficient, which in turn causes economic and environmental problems. Therefore, this work investigated lightweight geopolymer foams manufactured based on coal gangue, metakaolin, and a mix of them to evaluate the influence of such waste on the geopolymer matrix. In addition, the effect of 20% (wt.) of waste glass on the foams was determined. Mineralogical and chemical composition, thermal behaviour, thermal conductivity, compressive strength, morphology, and density of foams were investigated. Furthermore, the structure of the geopolymers was examined in detail, including pore and structure thickness, homogeneity, degree of anisotropy, porosity with division for closed and open pores, as well as distribution of additives and pores using micro-computed tomography (microCT). The results show that the incorporation of waste glass increased compressive strength by approximately 54% and 9% in the case of coal-gangue-based and metakaolin-based samples, respectively. The porosity of samples ranged from 67.3% to 58.7%, in which closed pores constituted 0.3–1.8%. Samples had homogeneous distributions of pores and additions. Furthermore, the thermal conductivity ranged from 0.080 W/(m·K) to 0.117 W/(m·K), whereas the degree of anisotropy was 0.126–0.187, indicating that the structure of foams was approximate to isotropic.

## 1. Introduction

A continuous increase in the global population is associated with growing housing needs and the demand for building materials [1]. Due to its ease of manufacture, low cost, versatility, and easy accessibility, concrete is the most commonly used construction material around the world [2]. However, it is noteworthy that the cement industry is responsible for emitting an enormous amount of carbon dioxide released during cement manufacturing, which constitutes 6–8% of total global anthropogenic emissions worldwide, even reaching 12% in China [3,4].

On the other hand, civilization progress and simultaneous technological development cause an increased demand for energy. Glushkov et al. [5] noticed that in recent years, energy consumption has grown by around 2–3% annually. Despite many efforts undertaken by the European Union countries, coal is still the most commonly used fossil fuel around the world [6]. In many countries, such as China [7], Australia [8], India [9], Poland [10], and the Czech Republic [11], this conventional energy source is one of the most significant constituents of domestic energy structure.

Coal gangue is a widespread inert solid waste developed from coal extraction, constituting about 15–20% of coal output, which in practice means that the production of 1 t of coal is associated with obtaining about 0.12 to 0.20 t of coal gangue [12]. Therefore, it is considered the most significant industrial type of waste in China, where over 6 billion tons of it is deponed annually [13]. Overall, coal gangue is mostly deposited, and therefore occupies a significant surface area on the one hand; on the other hand, it leads to soil degradation and the formation of geologic danger [14]. Many reports in the literature emphasised the capacity for combustion of coal gangue as a result of self-heating and reactions under air conditions, which is a threat to coal mines and all society as well due to the releasing of noxious gases into the atmosphere [15,16].

Another source is municipal waste, defined as waste of various origins, which is collected from households, factories, or institutional buildings. Their number is constantly growing due to economic development and the commonly accepted consumer lifestyle. One of them is waste glass, which constitutes 5.8% of all types of generated waste [17]. Glass is a well-known material, which is commonly used all over the world, and according to the literature data, every person consumes approximately 21 kg of glass per year [18]. Obviously, glass waste can be subjected to recycling and reusing, but statistics show that the global recycling rate reached only 21% [19]. The cause for this outcome lies in multiple colours of waste glass, which represent a crucial impediment in the recycling process and generates high costs due to the various chemical composition of each colour of waste glass. Furthermore, contamination occurring in the waste can also affect the chemical composition, hindering the recycling process [20]. Therefore, a huge amount of waste glass is constantly deposited, which in turn is connected with indispensability to bear the high costs.

Geopolymers constitute a group of modern and continuously developed materials, fabricated during the geopolymerisation process and more precisely through alkaline activation of silica-aluminous starting materials [21]. The geopolymer term was invented by Davidovits in 1972, who described them as aluminosilicate, characterised by a three-dimensional structure and formed as a result of activation by means of an alkaline solution [22,23]. Besides their environmental friendliness, geopolymers exhibit up-and-coming properties, proving the possibility of widespread prospective application [24]. Nevertheless, it should be highlighted that waste materials can be used for geopolymer production, such as construction and demolition waste [25], magnetic mining waste [26], brick waste [27], waste glass [28], and coal gangue [29].

Nowadays, geopolymer foams are applicable in a wide range of sectors of industries as thermal insulation of buildings, acoustic insulation, catalyst support, fire resistance, pH regulators, filters, and even flowerpots [30,31,32]. In general, one of the most popular methods for foam production relies on introducing chemical substances called foaming agents or surfactants into the material, which most often are aluminium powder, silicon powder, and hydrogen peroxide H_2_O_2_ [33]. However, it is worth mentioning that the properties of foams depend on many factors, such as type of base material [34] and its particle size [35], stabilizing agent [36], applied additive [37], curing conditions [38], and sintering application [39].

In recent years, the production of lightweight foam ceramics is becoming more and more popular in the scientific community. For instance, Zhang et al. [40] developed new glass-ceramic foams based on vitrified municipal solid waste incineration ashes, which were heated at 1150 °C for 2 h in the last stage of the manufacturing process. Researchers obtained materials characterised by porosity in the range of 79.23–88.35% and compressive strength of 0.36 to 5.55 MPa [40]. On the other hand, Li et al. [41] investigated glass-ceramic foams consisting mainly of recycled fluorite tailings and waste glass, which were produced at 1100 °C for 90 min. da Costa et al. [42] in their work applied waste glass, bentonite, and alumina in glass-ceramic foams, which were sintered at temperatures ranging from 750 °C to 800 °C, achieving porosity at the level of 52 to 85% and flexural strength of 0.2–3.7 MPa simultaneously. Overall, there is a lot of novel research focused on the application of waste glass in foams production, but the sintering process that requires high energy consumption and generates carbon dioxide is key in the great majority of them to obtain appropriate high porosity, low density, and at the same time relatively high mechanical properties [43,44,45]. However, it should be emphasised that the application of such high temperatures is connected with high energy consumption, which in turn negatively affects economic and ecological aspects. Therefore, it was shown that waste glass can also be used as an additive to the geopolymer matrix with the omitting of the sintering process [46]. Furthermore, it was proven that the most beneficial is to use waste glass with particle size below 75 µm due to the high pozzolanic reactivity; however, it also causes energy consumption related to its fragmentation [47].

Although it was proved that in general coal gangue is an appropriate raw material for geopolymer production, its utilization of it is still insufficient [48]. The possibility of waste application in the production of construction materials, which are characterised by an appropriate thermal conductivity, will be beneficial with regard to environmental protection. On the other hand, such a solution could have a positive effect on energy saving, especially concerning components intend for energy-saving buildings [49,50].

This work presented for the first time the possibility to obtain eco-friendly lightweight coal-gangue-based geopolymer foams reinforced with glass waste. Moreover, in order to compare the influence of metakaolin and coal gangue on the geopolymer properties, samples based on them both, as well as the metakaolin-based geopolymer, were thoroughly examined. The mineralogical and chemical composition, thermal conductivity, compressive strength, surface area, density, porosity, morphology, and thermal behaviour of foams were determined.

## 2. Materials and Methods

### 2.1. Materials

The coal gangue was received from the PG Silesia hard coal mine located in Czechowic-Dziedzice, Poland. The preparation of raw coal gangue included the following stages: preliminary disintegrating in a jaw crusher; grinding in the mill (Fritsch, Idar-Oberstein, Germany) to powder with particle size below 75 µm; and calcination at 800 °C for 24 h in a laboratory oven.

Metakaolin was bought from KERAMOST, Plc. company (Most, Czech Republic), whereas Portland cement with the commercial name CEM I 42,5R was purchased from Górażdże Cement S.A. company (Heidelberg Cement Group, Chorula, Poland).

Waste glass obtained from brown bottles was received from the local company Grabowski Export-Import (Sędziszowa, Poland), who crashed and initially cleaned them. However, there was no used additional cleaning; therefore, the bottles still contained contamination, such the remnants of labels. Furthermore, considering the energy-saving idea, the received waste glass did not undergo any additional milling.

A mixture consisting of 8 M sodium hydroxide solution and sodium silicate aqueous solution (R-145, ChemiKam, Będzin, Poland) mixed in a ratio of 1:2.5 was used as an alkaline solution. In geopolymer manufacturing, the liquid to solid ratio was established at 0.4.

The solution of hydrogen peroxide H_2_O_2_ (Chempur, Piekary Śląskie, Poland) with a concentration of 35% and density of 1.133 g/mol was used as a foaming agent.

### 2.2. Geopolymer Manufacturing Process

The first step in geopolymer manufacturing was mixing dry components, such as metakaolin, calcined coal gangue, cement, waste glass, and syringaldehyde, in order to obtain a homogeneous mix. The substitution level of waste glass was fixed based on a previous study, indicating that the optimal amount of this material in the geopolymer foams reached 20% by weight [51]. Commercially available cement with a high CaO content in the amount of 10% by weight was added to enhance pozzolanic reactiveness [52]. Moreover, syringaldehyde (chemical formula: HOC_6_H_2_(OCH_3_)_2_CHO) was added in the amount of 0.15% (wt.) as a stabiliser, which was selected based on the literature review [53].

Subsequently, the alkaline solution was added, and all components were mixed until the appropriate consistency of blends was acquired. The alkaline activator was prepared 24 h before use in pursuit to provide the entire course of the exothermic reaction and eliminate the possibility of fast setting because of heat hydration [54]. In the next step, the 35% solution of H_2_O_2_ in the amount of 3% (wt.) to the dry components was added. After that, the mixtures were poured into moulds and placed in a laboratory dryer (Chemland, Stargard, Poland) for 24 h at 75 °C. Geopolymers were tested after 28 days of curing at room temperature. Five types of geopolymer samples were investigated in this explorational work, and their compositions are given in Table 1.

### 2.3. Methods

Thermogravimetric analysis (TG/DTG) and Fourier-transform infrared spectroscopy (FT-IR) were performed using a TG-FTIR device NETZSCH TG 209F1 Libra equipped with an integrated FT-IR system (Erich NETZSCH GmbH & Co. Holding KG, Selb, Germany). Samples were heated in the temperature range from 25 °C to 1000 °C with a heating rate of 10 °C/min in an argon atmosphere. FTIR spectra were registered in the region of 600–4000 cm^−1^.

An X-ray fluorescence (XRF) spectrometer EDX-7200 (Shimadzu Corporation, Kioto, Japan) was applied to determine the chemical compositions of raw materials and geopolymers.

Particle Size Analyser PSA 1190 LD (Anton Paar, Graz, Austria) was utilised to determine the particle size distribution.

Determination of mineralogical composition was carried out using a Panalytical Aeris diffractometer (Malvern Panalytical, Almelo, The Netherlands). Measurements were recorded in the range 10° to 100° 2θ, applying Cu Kα radiation, time per step of 340, and step size of 0.003° (2θ). Subsequently, the results were analysed using the High Score Plus software version 4.8 (Panalytical) and the ICDD database (International Center for Diffraction Data, PDF4+). The quantitative analysis of mineral phases existing in raw materials and geopolymers were realised using the Rietveld refinement method.

Nitrogen adsorption–desorption measurements were conducted using Autosorb-iQ/MP Quantachrome Instruments (Anton Paar, Graz, Austria). The specific surface areas of investigated materials were determined by the Brunauer–Emmett–Teller (BET) method.

The density of the starting materials and geopolymers was assessed using a helium pycnometer Pycnomatic ATC Thermo Fisher Scientific (Waltham, MA, USA).

The morphological observation of geopolymers was performed by Keyence VHX-E100 digital microscope (Keyence, Osaka, Japan).

The thermal conductivity (λ) of geopolymers was explored using the heat flow meter HFM 446 Lambda Series (NETZSCH, Selb, Germany). Measurements were carried out in 3 different temperature ranges: 0–20 °C, 20–40 °C, and 30–50 °C, in accordance with DIN EN 12667 standard [55].

The compressive strength of geopolymers was evaluated after 28 days of curing in room conditions using the MTS Criterion Model 43 device (MTS Systems, Eden Prairie, MN, USA) in accordance with EN 12390:2019 standard [56], and each type of material was tested using at least 3 cubic samples.

Examination of geopolymers by means of X-ray micro-computed tomography was performed using Phoenix nanotom s (Skaneateles, NY, USA). Quantitative analysis of obtained data was carried out using the Fij open-source software version 1.53f51 and Bone J plugin intended for the investigation of porous structures. The central part of the specimens was used as a region of interest (ROI) for the analysis with dimensions of approximately 27 mm × 27 mm × 27 mm.

## 3. Results and Discussion

### 3.1. Raw Materials

**Figure 1:**
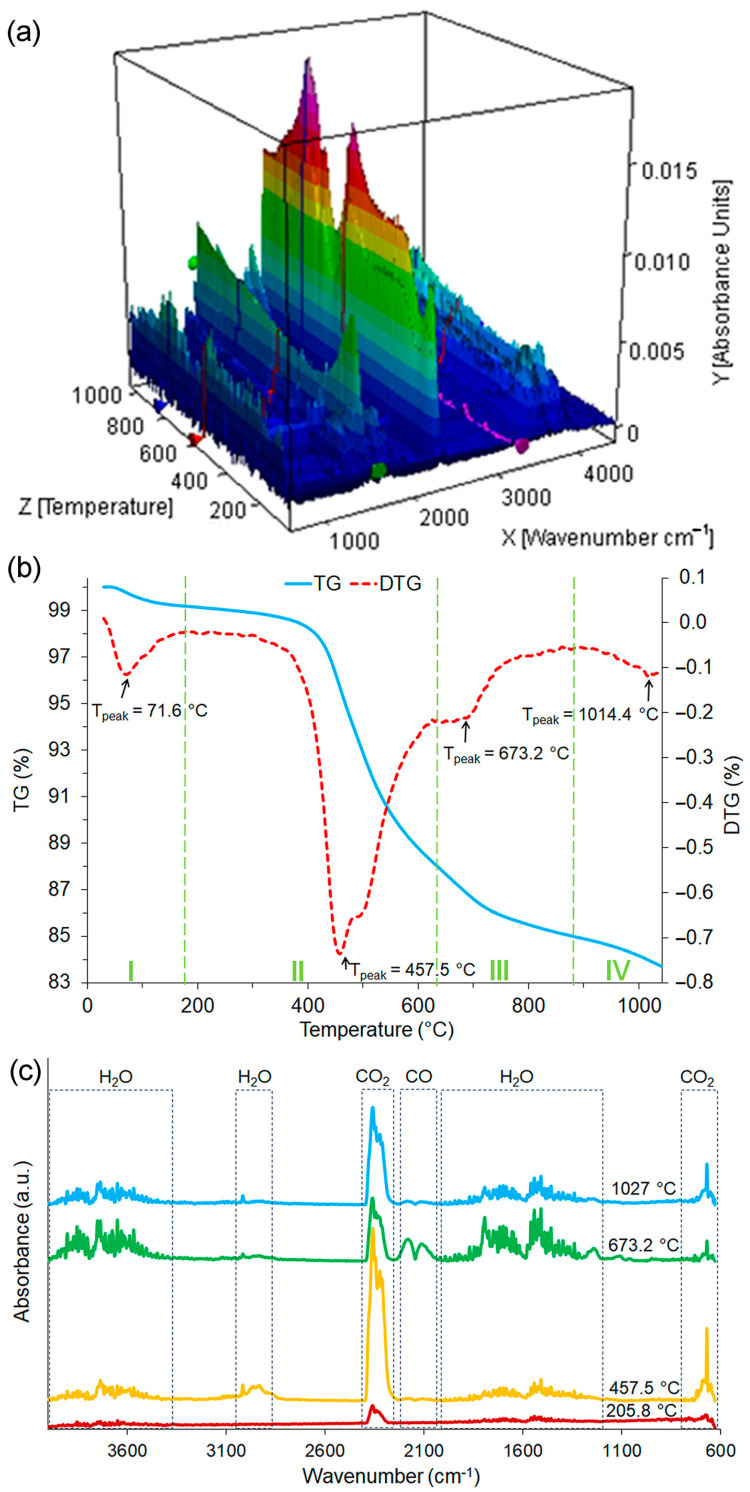
Thermal analysis results of coal gangue: (**a**) the 3D TG-FTIR spectra of emitted gas products; (**b**) TG and DTG curves; (**c**) 2D TG-FTIR spectra of gas products for representative temperatures.

The raw crushed coal gangue was examined using TG–FTIR thermogravimetric analysis to determine the optimal calcination temperature and explore the gas products emitted during the heating. The 3D surface plot for TG-FTIR spectra of released gases, the thermogravimetric curve (TG) and its first derivative (DTG), and the 2D FTIR spectra for distinctive temperatures are shown in Figure 1a–c, respectively. On the basis of the obtained three-dimensional graph (Figure 1a), it is evident that the intensities of individual absorption bands changed depending on the temperature. The majority of gas products were released in the temperature range of about 200 °C to 1000 °C, and this was in line with the course of TG-DTG curves (Figure 1b). In general, the weight loss of coal gangue was a four-stage process, as marked on the diagram. These phenomena occurred in the following temperature ranges: from room temperature to 186 °C, 186 to 631 °C, 631 to 864 °C, and 86 to 1049 °C, and that corresponded with mass losses of 0.83%, 11.10%, 2.97%, and 1.48%, respectively. The first stage was connected with the dehydration process of water and desorption of gases trapped on the coal gangue surface, and it can be seen that the maximal effect of these was registered at 71.6 °C. After that, the oxidation process started with the maximum achieved at 205.8 °C [57]. Furthermore, the most significant change in mass was registered at the temperature between 186 and 631 °C, with the maximum temperature peak at 457.7 °C in the DTG curve, which occurred as a result of dehydroxylation of kaolinite and the constituting of metakaolinite [58]. However, it should be noted that after that stage, two consecutive smaller mass loss steps were observed at the temperature ranges of 631–864 °C and 863–1049 °C. They could be attributed to the combustion process, including the decomposition of organic matter [59]. The residual mass of coal gangue registered at the end of the measurement at 1050 °C was 83.62%. As shown in Figure 1c, all of the FTIR spectra corresponding to the characteristic temperatures consisted mainly of CO_2_, H_2_O, and CO, and this is compliant with the previous work [60]. The difference in the intensity of the spectra obtained for individual samples can be explained by the amount of gas released at a specific temperature.

On the basis of the described result, 800 °C was fixed as an optimal calcination temperature of coal gangue. In general, metakaolin can be obtained below this temperature; nonetheless, this choice was made due to the necessity to remove carbon from the raw material dedicated to geopolymer manufacturing.

The chemical composition of raw materials is presented in Table 2. Coal gangue in both stages (before and after calcination) and metakaolin had a quite similar composition, with two main compounds, SiO_2_ and Al_2_O_3_, which is consistent with previous research [61,62]. The difference between these materials was the Fe_2_O_3_ content, which was much higher in the case of coal gangue compared to metakaolin. On the other hand, ordinary Portland cement contained CaO in the most enormous quantity, and simultaneously it had about content of SiO_2_ that was three times lower than that of coal gangue and metakaolin. Moreover, taking into account the effect of the used treatment on the coal gangue, it can be concluded that the calcination process resulted in an increase in Fe_2_O_3_ and reduction in SO_3_ content. The summary content of SiO_2_, A1_2_O_3_, and Fe_2_O_3_ amounted to 90.168%, 96.680%, and 97.76% for raw coal gangue, calcinated coal gangue, and metakaolin, respectively, and consequently, they can be applied as cement material [63].

**Figure 2:**
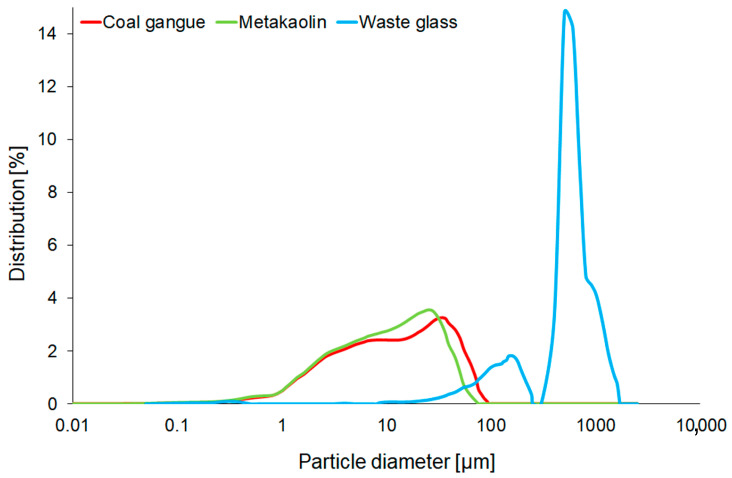
The particle size distribution of waste glass, metakaolin, and coal gangue before the calcination process.

The particle size distribution curves for raw materials are presented in Figure 2, which points out that metakaolin consisted of the smallest particle size of all investigated materials. However, the course of obtained curves was fairly similar in the case of metakaolin and coal gangue, and it consisted of the peak with top constituting particles with sizes of 25 µm and 32 µm for metakaolin and coal gangue, respectively. In contrast, the curve for waste glass has a bimodal distribution, suggesting that it consisted of particles with a more significant variation than the previously discussed materials.

Table 3 shows the particle size distribution parameters for all starting materials. The influence of particle size on the properties of the coal-gangue-based geopolymer was earlier examined by Li et al. [64], and they concluded that the optimal particle size was obtained by means of grinding and sieving using 200 mesh (74 µm), and the achieved average particle size (D_50_) was 10.79 μm. Similarly, in the presented work, D_50_ of coal gangue amounted to 11.17 µm, and it was measured before calcination. However, for metakaolin, a lower value of D_50_ (9.60 μm) by approximately 16% was registered. On the other hand, waste glass was characterised by the most enormous particle size (D_50_ 474.9 µm), but the calculated span was small (1.6 µm).

**Figure 3:**
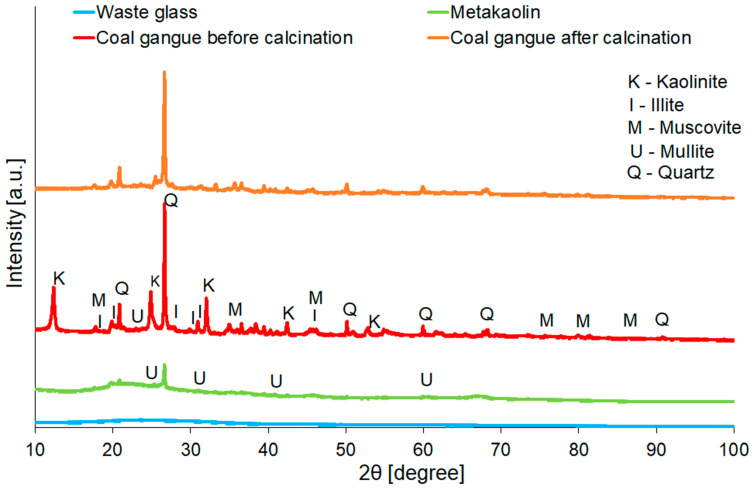
XRD patterns of waste glass, metakaolin, and coal gangue before and after calcination.

The XRD patterns of raw materials are demonstrated in Figure 3, whereas the results of the quantitative analysis are summarised in Table 4. However, it is worth noticing that the presented results of the quantitative analysis were estimated due to the occurrence of an amorphous phase, which was visible as a diffuse halo between 15° and 40° 2θ on the diagram [65]. As a result of the qualitative analysis of coal gangue, the following phases were identified: quartz (ICDD card numbers: 01-075-8320); Kaolinite-1Ad (ICDD card numbers: 01-078-2110); Illite-2M1 (ICDD card numbers: 00-026-0911); and Muscovite-2M1 (ICDD card numbers: 00-006-0263). In the comparison of the results of coal gangue before and after treatment, it was noticed that the difference was in the contents of kaolinite and quartz. These findings were consistent with the other studies [66,67]. The kaolinite was still detectable in very low quantities after calcination of coal gangue, indicating that applied treatment did not cause entire dehydroxylate kaolinite, but it was still highly efficient as expected. Increasing the content of quartz (SiO_2_) was also beneficial in terms of the geopolymerisation process [68]. Moreover, mullite was detected in the metakaolin structure, which existed as a result of high-temperature treatment [69].

Furthermore, the true density of raw materials was examined, and it achieved 2.566 ± 0.001 g/cm^3^ for metakaolin, 2.507 ± 0.001 g/cm^3^ for waste glass, 2.273 ± 0.001 g/cm^3^ for coal gangue before treatment, and 2.821 ± 0.001 g/cm^3^ for calcinated coal gangue.

**Figure 4:**
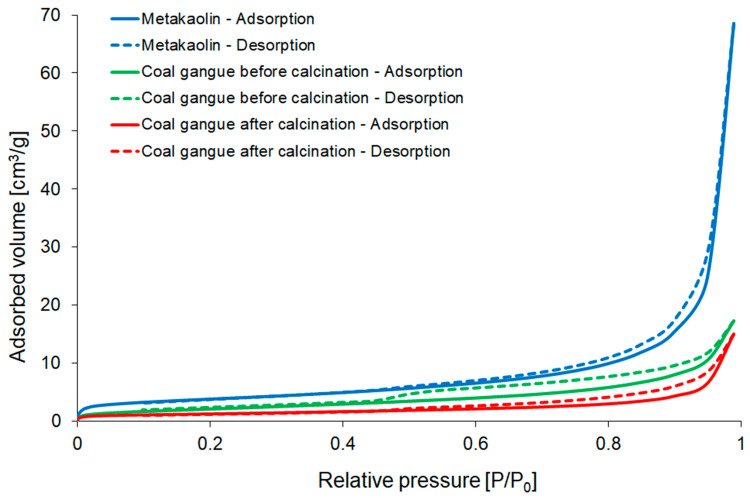
Nitrogen adsorption–desorption isotherms of metakaolin and coal gangue before and after calcination.

The obtained N_2_ adsorption–desorption isotherms of raw materials are shown in Figure 4. According to IUPAC (International Union for Pure and Applied Chemistry) division, all of them can be classified as type IV, which is representative of mesoporous adsorbents [70]. Hysteresis loops enable the determination of the shape of pores occurring in the investigated material, and according to the classification, they exhibited H3 type, indicating the presence of slit-shaped pores. On the other hand, coal gangue in both stages showed H3-type hysteresis, which is attributed to slit-shaped pores [71].

The specific surface area of metakaolin determined by means of the multi-BET method equalled 13.37 m^2^ g^−1^ (Table 5), which is consistent with other studies [72,73]. The calcination process of coal gangue decreased hysteresis loops and specific surface area (by around 50%) and slightly reduced pore volume.

### 3.2. Geopolymers

**Figure 5:**
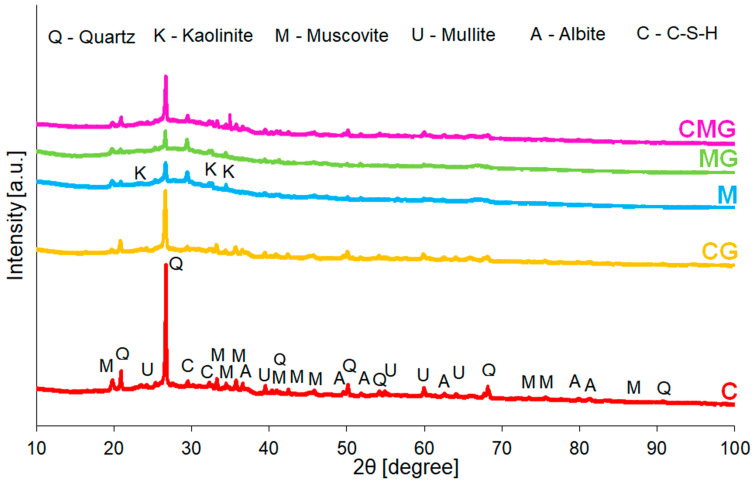
XRD patterns of geopolymer foams.

On the basis of the results of the diffraction pattern shown in Figure 5, the mineral composition of geopolymers consisted of quartz, kaolinite, muscovite, mullite, albite, and C-S-H existing in the form of rosenhanite. Different from the raw material, all of the geopolymers contained mullite, albite, and a C-S-H phase. Moreover, the characteristic diffraction peak derived from quartz at 26.6° 2θ had the highest intensity in the case of the coal-gangue-based sample (C). Cheng et al. [74] suggested that the reduction in its intensity can be associated with a beneficial impact on compressive strength and therefore it can be assumed that the addition of waste glass, as well as metakaolin, was profitable. Furthermore, it can be seen that the intensity of quartz decreased with increasing content of metakaolin raw material. The quantitative analysis of coal-gangue-based geopolymer foams did not detect the kaolinite phase, which confirmed that during treatment the inner hydroxyl structure of kaolinite was reduced, and as a result, an amorphous substance material was formed [75]. Qualitative analysis of geopolymer (Table 6) showed new phases, which were albite and C-S-H, proving the reaction between raw materials and alkaline activator during geopolymerisation [76]. Similarly, results also revealed the mullite phase in all foams, whereas it was identified only in metakaolin from among raw materials. In addition, obtaining C-S-H gel, which is characterised by a dense structure, was beneficial due to the possibility to increase the strength of geopolymers [77]. Furthermore, Table S1 in the Supplementary Materials shows the influence of waste glass, coal gangue, and metakaolin on the chemical composition of samples. X-ray fluorescence confirmed that geopolymers mainly consisted of SiO_2_, Al_2_O_3_, Fe_2_O_3_, and CaO regarding chemical composition. According to the results, the incorporation of waste glass slightly changed the content of SiO_2_ in foams, as expected. On the other hand, the Al_2_O_3_ content, which is crucial in terms of geopolymerisation process, was higher in metakaolin-based samples.

**Figure 6:**
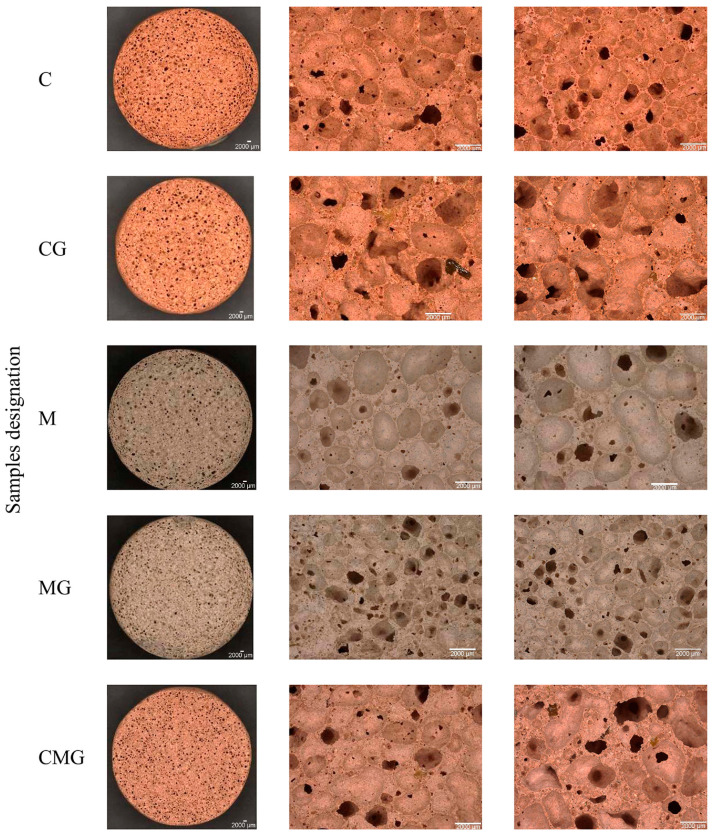
Optical micrographs of geopolymer foams.

Representative structures of the produced foams observed under an optical microscope are shown in Figure 6. The porous structure of geopolymers was formed as a result of the application of hydrogen peroxide, which exhibits thermal instability in basic media and then decomposes according to equations the following: H_2_O_2_ + OH^−^ ⟶ HO_2_^−^ + H_2_O and subsequently HO_2_^−^ + H_2_O_2_ ⟶ H_2_O + O_2_ + OH^−^ [78,79]. Based on observation, it was noted that metakaolin-based samples contained smaller pores than their coal-gangue-based counterparts. Unreacted particles of waste glass are visible in the pore structure, which can lead to a decrease in mechanical properties. The pore distribution was relatively homogenised in foams; however, coal-gangue-based samples included voids heading along the height, and their shape is more irregular than that of samples containing metakaolin. In general, the macropores were visible in examined samples, and the pore distribution was quite homogeneous for each type of foam. It should be noted that slightly different pore shapes were around the edges of the sample, and this can be explained by boundary conditions and the influence of the applied mould [80].

Moreover, the conducted observations revealed that small inconsistencies, seemingly cracks, were observed in the coal gangue samples. This phenomenon can explain that the geopolymerisation process, in the case of using coal gangue, was not as effective as with using metakaolin.

**Figure 7:**
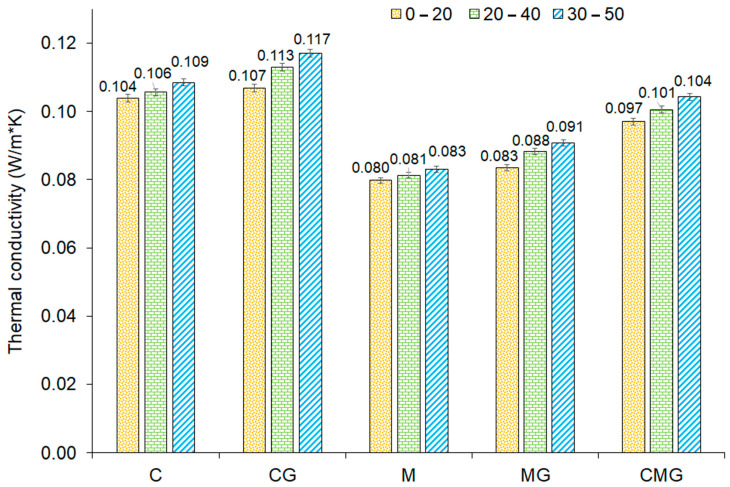
Thermal conductivity of geopolymer foams determined at different temperature ranges.

Thermal conductivity measurements were conducted in three various temperature ranges, namely 0–20 °C, 20–40 °C, and 30–50 °C, which are demonstrated in Figure 7. In general, the thermal coefficient (λ) of geopolymers was in the range of 0.079–0.117 W/(m·K). The thermal conductivity increased with the rising temperature, which is a well-known dependence for insulators [81]. The difference in results in thermal conductivity depending on complied temperature ranged up to 9%, which is visible in the diagram. Moreover, it was found that the thermal coefficient increased with the addition of waste glass. Furthermore, metakaolin-based samples showed lower values of the thermal coefficient than foams manufactured using coal gangue. It is well known that the thermal conductivity of geopolymer foams is dependent on the porosity of samples. The increase in sample porosity results in obtaining lower thermal conductivity. Based on the morphology analysis, it can be stated that pores can be open or closed. There was a tendency of closed-cell foams to demonstrate a lower value of the thermal coefficient than open-cell foams at a similar density [82]. For example, Smiljanić et al. investigated the thermal conductivity of waste glass, and they obtained λ coefficients of 1.30 W/(m·K) and 1.28 W/(m·K) for powder in the delivery condition (D_50_ 17 μm) and after 35 min of milling (D_50_ 8 μm) [83]. The particle size of the waste glass was too high to dissolve completely in the geopolymer matrix, and this was also confirmed by microscopy analysis. Therefore, it can be assumed that for the part quantity of added waste glass, thermal conductivity was close to the state of delivery and thus higher than for the geopolymer matrix without an additive.

**Figure 8:**
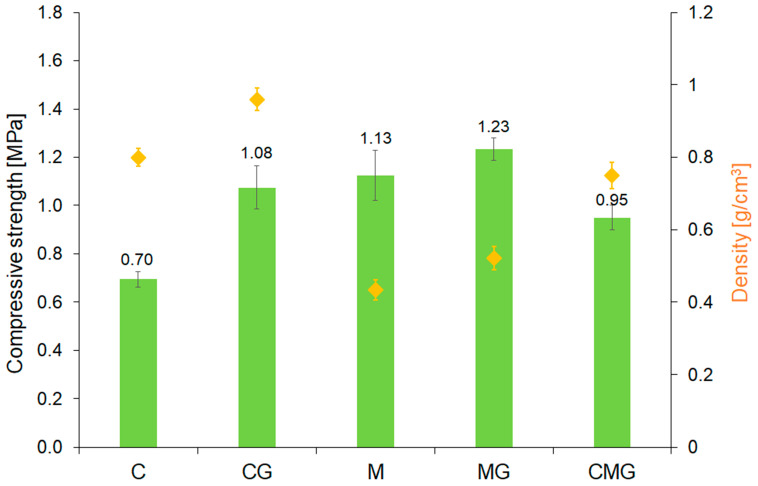
Compressive strength of geopolymer foams.

Figure 8 displays the density and results of the compressive strength test of geopolymer foams. It is evident that the incorporation of waste glass had a positive and significant effect on the mechanical properties. Geopolymers reinforced by waste glass exhibited approximately 54% and 9% higher compressive strength in the case of coal-gangue-based and metakaolin-based samples, respectively, compared to samples without the addition. This visible reduction in the density of foams suggests that the structure pores have changed, which in turn had an impact on the physical and mechanical properties, such as compressive strength. Moreover, a dependence between the compressive strength and the density of foams was found. The waste glass addition resulted in a higher density of geopolymers as a result of the reduction in pore volume and simultaneously positively affected the compressive strength. It should be noted, however, that the metakaolin used as the base material had a positive effect on both reducing the density and increasing the compressive strength. This phenomenon may be the result of the better homogeneity of the structure of the metakaolin-based foams.

**Figure 9:**
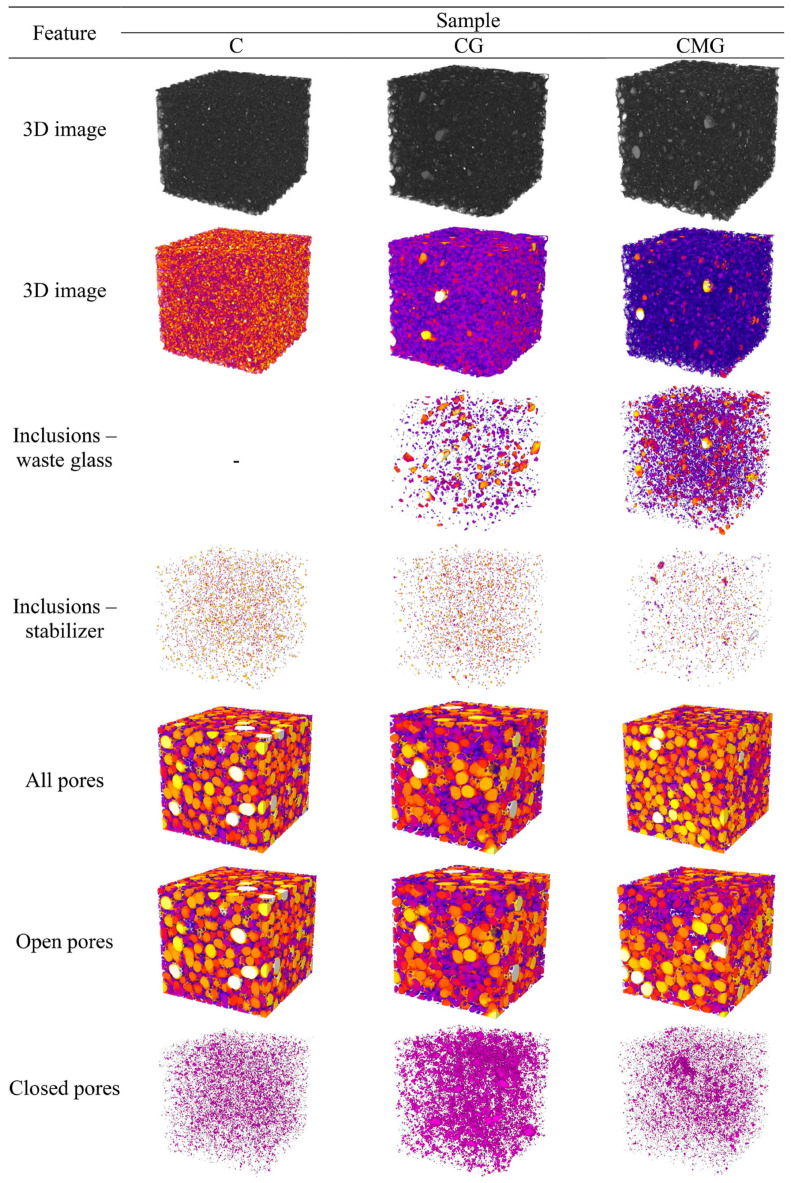
X-ray micro-computed tomography of geopolymers, showing individual features of foams.

In order to evaluate the structural properties of the geopolymer, representative samples were selected and investigated using computer microtomography. The selection criterion was the content of coal gangue in the sample to evaluate the influence of metakaolin first, followed by waste glass. The individual characteristic features of the representative samples of the coal gangue, metakaolin, and the mix of them are presented in graphical form in Figure 9. Moreover, Table S2 in the Supplementary Materials presents a 3D view of the sample, a 2D view of the sample (first slice) with a yellow line, and the slice perpendicular to the marked yellow line.

Furthermore, quantitative analysis of geopolymer foams was performed using Fiji software. The following features were measured: porosity (including open and closed pores); inclusions (waste glass designated as WG and stabiliser (syringaldehyde) designed as S); structure thickness; pores thickness; degree of anisotropy; and homogeneity (considering three directions: XY, XZ, and YZ and an average value of them). Obtained results are summarised in Table 7.

The porosity of the samples ranged from 69.0% to 58.7%. Comparing the influence of coal gangue and metakaolin, it can be concluded that a more porous structure was obtained using metakaolin as a raw material. Similarly, replacing 20% (wt.) of coal gangue with waste glass generated lower porosity of the geopolymers. In all analysed samples, open pores dominated the material structure, achieving 68.7%, 56.8%, and 66.9% for C, CG, and CMG, respectively. In general, two types of inclusions were detected in geopolymers, except for the C sample, which was consistent with the geopolymer manufacturing procedure, because only this sample did not include waste glass. The greatest results of structure thickness, as well as pores thickness were measured for the CG sample, which suggests that this material had the largest pores and, at the same time, the thickest wall structure. The smallest size of pores was attributed to the ample manufactured based on a mix, to wit coal gangue, metakaolin, and waste glass.

It is well known that the degree of anisotropy reaches 0 value for a fully isotropic structure, whereas a calculated value of 1 means that the structure is anisotropic. Therefore, it can be concluded that foams had structures approximate to isotropic. Moreover, the addition of waste glass to the geopolymer resulted in a lower degree of anisotropy, and this effect was also strengthened by introducing metakaolin. The homogeneity of foams was investigated in three directions, and it is certain that all fabricated samples had homogeneous structures, as can be seen from almost identical values of the obtained results.

**Figure 10:**
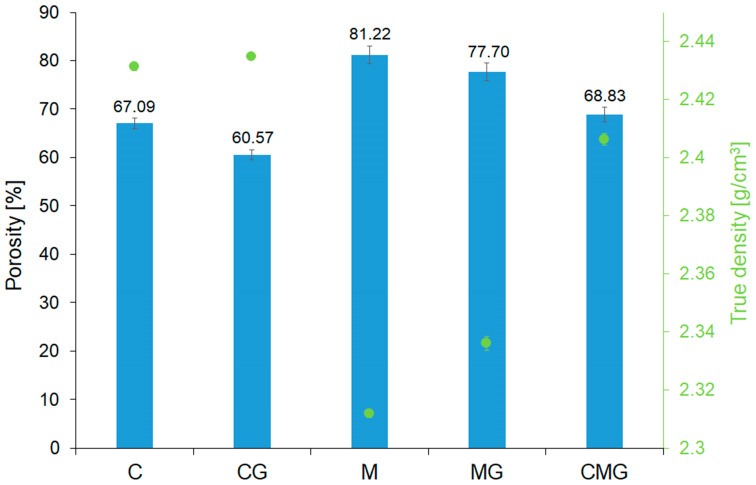
Comparison between the porosity and true density of the geopolymer foams.

In order to compare the porosity of all samples, porosity was calculated based on their apparent densities and true density in accordance with the formula: Porosity = 1 – (apparent density − true density) [84]. In Figure 10, the obtained results of porosity, as well as true density, are demonstrated. It can be noticed that the results obtained by micro-computed tomography and those determined on the basis of density measurements were highly consistent. Similar observations were made by other authors [85]. The highest porosity had metakaolin-based samples (M), as expected. Subsequently, the addition of 20% (wt.) of waste glass into metakaolin-based samples (MG) resulted in a decrease in porosity by around 5% compared to the designed M samples. It is clearly visible that using coal gangue resulted in lower porosity of geopolymer foams compared to metakaolin. However, the porosity tended to decrease with the addition of waste glass, regardless of the applied geopolymer raw material.

Due to the lack of standards intended for geopolymers, the application of standards for concrete is a common practice [86]. In general, foam concrete is defined as a lightweight material that incorporates air voids. On the other hand, it is characterised by appropriate properties, such as density, thermal insulation, strength, and composition [87]. Based on the obtained results, it was found that the presented materials, with only one exception for CG due to the higher density, can be classified as lightweight concretes class III according to the division of the International Union of Laboratories and Experts in Construction Materials, Systems and Structures (RILEM). This means that foams comply with the following requirements: Lambda coefficient in the range of 0.065–0.22 W/(m·K); compressive strength between 0.7 MPa and 3.4 MPa; and a density of 240–800 kg/m^3^. Moreover, such materials were determined as insulating lightweight concrete [88,89].

## 4. Conclusions

In this work, the results of research focusing on eco-friendly, low-cost geopolymer foams characterised by low thermal conductivity are presented. Calcinated coal gangue and metakaolin were used as the starting materials; furthermore, waste glass was applied as an additive. Based on the obtained results, it was found that manufactured materials exhibited excellent potential for applications as insulating materials, for instance, of walls, roofs, and ceilings. Furthermore, the main gaseous products that evolved during the heating of the raw materials were CO_2_, H_2_O, and CO. Coal gangue in both stages (before and after calcination) and metakaolin had a quite similar composition, with two main compounds, namely SiO_2_ and Al_2_O_3_, indicating that they can be used as geopolymer precursors. Metakaolin-based samples contained smaller pores than their coal-gangue-based counterparts. Pore distribution is relatively homogenised in foams, but notably, coal-gangue-based samples included voids heading along the height, and their shape is more irregular than in samples containing metakaolin. In addition, small inconsistencies, seemingly cracks, were observed in coal-gangue-based samples, which had a negative impact on their compressive strength. The thermal conductivity of geopolymers was dependent on the porosity of samples, indicating that the increase in porosity resulted in lower thermal conductivity. However, the addition of coal gangue, as well as waste glass, resulted in an increased thermal conductivity coefficient. Geopolymers reinforced by waste glass exhibited approximately 54% and 9% higher compressive strength in the case of coal-gangue-based and metakaolin-based samples, respectively, compared to samples without the addition. The porosity of the samples ranged from 58.7% to 67.3%, and closed pores constituted only 0.3–1.8% of it. Comparing the influence of coal gangue and metakaolin, it can be concluded that a more porous structure was obtained using metakaolin as a raw material. Moreover, the foams, which can be classified as lightweight concretes class III, had structures approximate to isotropic and they showed simultaneous homogeneous distribution of pores and additives. The presented research revealed the possibility of waste management from the mining industry in the building sector. Application of this type of materials complies with the circular economy concept and therefore should be developed in the future, especially on an industrial scale.

## Figures and Tables

**Table 1:** Mix design of geopolymer samples.

Samples Designation	Coal Gangue (%)	Metakaolin (%)	Cement (%)	Waste Glass (%)
C	90	-	10	-
CG	70	-	10	20
M	-	90	10	-
MG	-	70	10	20
CMG	35	35	10	20

**Table 2:** Chemical composition of raw materials used for geopolymer production.

Compound Formula	Raw Material				
	Coal Gangue before Calcination	Coal Gangue after Calcination	Metakaolin	Cement	Waste Glass
	Content (%)				
SiO_2_	56.540	53.501	54.851	18.339	81.355
Al_2_O_3_	26.180	26.683	41.841	3.673	1.587
Fe_2_O_3_	7.448	12.496	1.068	4.524	1.030
K_2_O	3.898	3.092	1.163	0.745	0.703
SO_3_	2.437	0.878	0.076	4.202	0.003
CaO	1.677	1.325	0.426	67.745	14.929
TiO_2_	1.410	1.179	0.309	0.293	0.084
MnO	0.078	0.120	-	0.202	0.068
V_2_O_5_	0.075	0.055	0.016	0.017	
P_2_O_5_	0.062	0.515	0.097	-	
SrO	0.044	0.038	0.011	0.130	0.035
Cr_2_O_3_	0.033	0.042	-	0.036	0.070
ZrO_2_	0.030	0.025	0.010	0.010	0.028
ZnO	0.019	0.016	0.006	0.055	0.007
SnO_2_	0.014	0.012	-	-	
CuO	0.011	0.010	0.004	0.024	0.007
Y_2_O_3_	0.010	0.008	0.002	0.002	0.001
BaO	-	-	-	-	0.079

**Table 3:** Particle size distribution parameters of waste glass, metakaolin, and coal gangue before the calcination process.

Material	D_10_	D_50_	D_90_	Mean Size	Span (D_90_ − D_10_)/D_50_
	[µm]	[µm]	[µm]	[µm]	[µm]
Coal gangue	1.82 ± 0.05	11.17 ± 0.36	44.32 ± 2.09	18.85 ± 0.81	3.80 ± 0.07
Metakaolin	1.63 ± 0.02	9.60 ± 0.12	32.50 ± 0.81	14.55 ± 0.29	3.21 ± 0.04
Waste glass	111.4 ± 11.7	474.9 ± 18.6	885.2 ± 66.5	541.1 ± 15.30	1.6 ± 0.2

**Table 4:** Quantitative phase analysis of metakaolin and coal gangue before and after calcination.

Raw Material	Identified Mineralogical Compound (%)				
	Quartz	Kaolinite-1Ad	Illite-2M1	Muscovite-2M1	Mullite
	SiO_2_	Al_2_Si_2_O_5_(OH)_4_	(KH_3_O)Al_2_Si_3_AlO_10_(OH)_2_	KAl_2_(Si_3_Al)O_10_(OH,F)_2_	Al_6_O_5_(SiO_4_)_2_
Coal gangue before calcination	27.4	49.5	11.5	11.5	-
Coal gangue after calcination	57.1	0.1	21.4	21.4	-
Metakaolin	6.3	48.0	20.6	20.6	4.6

**Table 5:** Specific surface area, pore size, and volume of metakaolin and coal gangue before and after calcination.

Material	Specific Surface Area (m^2^ g^−1^)		Pore Volume (cm^3^ g^−1^)		Pore Size (nm)
	Single-Point BET	Multi-Point BET	BJH Pore Volume	Total Pore Volume at P/P_0_ = 0.99	BJH Average Pore Diameter
Coal gangue before calcination	6.476	8.043	0.027	0.027	2.453
Coal gangue after calcination	3.819	4.150	0.023	0.023	3.056
Metakaolin	12.590	13.370	0.106	0.106	4.317

**Table 6:** Phase composition of investigated geopolymers.

Sample	Identified Mineralogical Compound					
	Quartz	Kaolinite	Muscovite	Mullite	Albite	CSH
	SiO_2_	Al_2_Si_2_O_5_(OH)_4_	KAl_2_(Si_3_Al)O_10_(OH,F)_2_	Al_6_O_5_(SiO_4_)_2_	NaAlSi_3_O_8_	Ca_3_Si_3_O_9_·H_2_O
C	27.9	0.0	35.4	10.1	0.2	26.4
CG	22.5	0.0	29.5	11.8	11.3	24.9
M	3.0	0.3	41.2	4.2	21.0	30.3
MG	2.6	0.6	58.9	3.0	16.8	18.0
CMG	9.7	1.0	21.7	9.7	25.0	32.9

**Table 7:** Characteristic parameters of geopolymer foams, calculated based on the X-ray micro-computed tomography results.

Sample	Porosity			Inclusions		Structure Thickness	Pores Thickness	Degree of Anisotropy	Homogeneity			
	Total [%]	Open [%]	Closed [%]	WG [%]	S [%]	[mm]	[mm]	-	XY [CTN]	XZ [CTN]	YZ [CTN]	Mean [CTN]
C	69.0	68.7	0.3	-	0.31	0.30 ± 0.08	1.62 ± 0.732	0.187	78.5 ± 15.4	78.5 ± 17.1	78.5 ± 16.3	78.5 ± 16.3
CG	58.7	56.8	1.8	1.08	0.26	0.62 ± 0.20	1.83 ± 0.658	0.156	104.9 ± 21.5	104.9 ± 21.3	104.9 ± 22.7	104.9 ± 21.8
CMG	67.3	66.9	0.4	3.74	0.21	0.33 ± 0.18	1.39 ± 0.569	0.126	82.9 ± 16.2	82.9 ± 17.1	82.9 ± 16.6	82.9 ± 16.6

## Data Availability

Not applicable.

## Supplementary Materials

The following supporting information can be downloaded at: https://www.mdpi.com/article/10.3390/ma16176054/s1, Table S1: Chemical composition of geopolymer foams; Table S2: 3D view of the sample, 2D view of the sample (first slice), slice perpendicular to the marked yellow line.
