# Supplementary material for: Eco-Friendly Coal Gangue and/or Metakaolin-Based Lightweight Geopolymer with the Addition of Waste Glass
Source: Materials (Basel). 2023 Sep 3;16(17):6054. doi: 10.3390/ma16176054 (PMC10489159; doi:10.3390/ma16176054)
Supplement: Supplementary file 1 [file materials-16-06054-s001.zip › materials-2570298-supplementary.pdf]

materials

Supplementary material

Eco-Friendly Coal Gangue and/or Metakaolin-Based Lightweight Geopolymer with the Addition of Waste Glass

Celina Ziejewska [1], Agnieszka Bąk [1], Krzysztof Hodor [2] and Marek Hebda [1,*]

[1] Cracow University of Technology, Faculty of Materials Engineering and Physics, Warszawska 24, 31-155, Kraków, Poland; celina.ziejewska@pk.edu.pl (C.Z.); agnieszka.bak@pk.edu.pl (A.B.)
[2] NETZSCH (Netzsch Instrumenty Sp. z o.o.), Halicka 9, 31-036 Kraków, Poland; krzysztof.hodor@netzsch.com (K.H.)
* Corresponding: marek.hebda@pk.edu.pl (M.H.)

Table S1. Chemical composition of geopolymer foams.

Chemical compound	C	CG	M	MG	CMG
SiO_2	55.957	56.680	61.012	61.026	61.299
Al_2O_3	18.075	14.622	28.684	27.562	21.301
Fe_2O_3	11.654	11.797	1.650	1.596	5.350
CaO	8.067	11.189	6.356	7.654	8.466
K_2O	2.823	2.700	1.253	1.139	1.690
SO_3	1.906	1.520	0.485	0.497	1.059
TiO_2	1.135	1.075	0.372	0.345	0.600
MnO	0.126	0.140	0.033	0.038	0.066
V_2O_5	0.065	0.061	0.021	0.017	0.033
SrO	0.061	0.063	0.027	0.029	0.041
Cr_2O_3	0.035	0.040	0.011	0.010	0.018
ZrO_2	0.033	0.034	0.016	0.016	0.025
ZnO	0.026	0.028	0.013	0.012	0.016
CuO	0.015	0.014	0.008	0.007	0.009
Ga_2O_3	0.007	-	0.010	0.007	0.007
NbO	0.003	-	0.003	0.002	0.002

Table S2. 3D view of the sample, 2D view of the sample (first slice), slice perpendicular to the marked yellow line.

Samples designation			
C	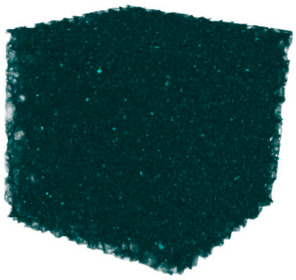	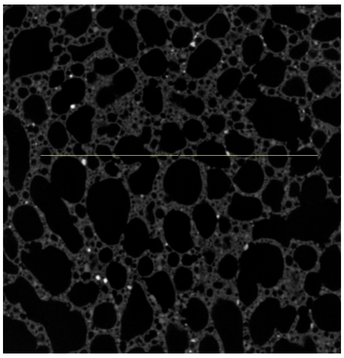	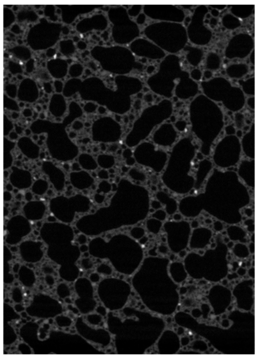
CG	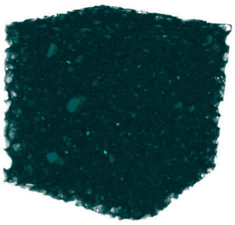	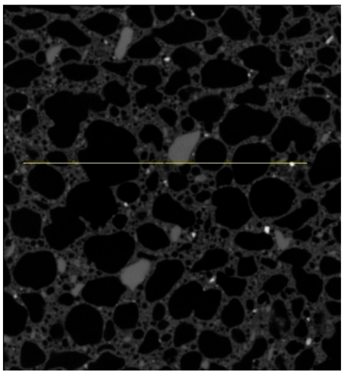	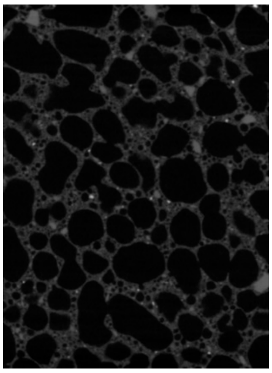
CMG	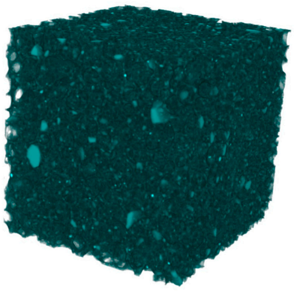	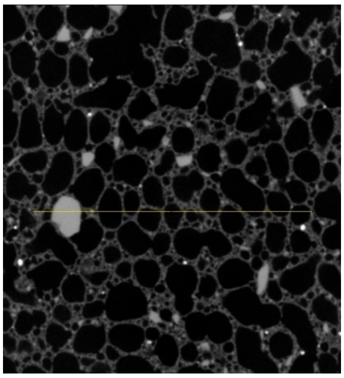	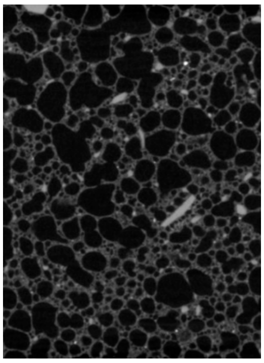
